# Consequences of COVID-19 Lockdown Restrictions on Children Physical Activity—A Slovenian Study

**DOI:** 10.3389/fpubh.2022.843448

**Published:** 2022-03-10

**Authors:** Jurij Planinšec, Črtomir Matejek, Saša Pišot, Rado Pišot, Boštjan Šimunič

**Affiliations:** ^1^Faculty of Education, University of Maribor, Maribor, Slovenia; ^2^Science and Research Centre Koper, Institute for Kinesiology Research, Koper, Slovenia

**Keywords:** COVID-19 pandemic, physical activity, children, remote schooling, physical education

## Abstract

During the COVID-19 pandemic, countries took several restrictions to contain the spread of coronavirus. In the second wave of the COVID-19 pandemic, primary schools in Slovenia were closed for a period long time (from October 19th 2020 until January 18th 2021 when they were partially reopened for 6–9 year olds until February 15th 2021 when they were reopened for all children) and organized sport activities for children and adolescents under the age of 15 was not allowed during this period. The aim of the study was to examine how these restrictions were reflected in the amount of different forms of physical activity (PA) of 6–12-year old children (*N* = 3,936). Data were collected using an online questionnaire (International Physical Activity Questionnaire Short Form) comparing different forms of PA before (BEFORE) and during (DURING) remote schooling. The results show that there has been a decline in children's PA DURING, specifically, only 4.3% of children had their physical education ≥ 45 min (or 77.7% ≤ 30 min), as is the usual duration in Slovenia. There was also a remarkable decline in extracurricular sports activities (*p* < 0.001), which BEFORE had been participated by 72.2% of children, while DURING remote schooling, as many as 83.5% of children did not participate these activities. 69.7% of children participated in organized sports in clubs at least once a week, while DURING remote schooling, as many as 88.1% (*p* < 0.001) did not participate in such form of activities. Furthermore, the time spent exercising in moderate to vigorous PA also decreased (BEFORE 8.2% vs. DURING 24.9%; *p* < 0.001). We found that during lockdown there has been an alarming decrease in the frequency and duration of organized PA at school and at sports clubs. These findings are a good starting point for designing (developing) an effective strategy for promoting health-enhancing PA of children in the event of a future lockdown or similar situations. The strategy should focus on the appropriate implementation of PA curriculum and motivate young people to participate regularly in extracurricular organized and non-organized activities.

## Introduction

The COVID-19 pandemic, public health recommendations and governmental measures have enforced lockdowns and restrictions. While these restrictions help to abate the rate of infection, such limitations result in negative effects by limiting participation in normal daily activities, physical activity (PA), travel and access to many forms of exercise (e.g., closed gyms, no group gatherings, increased social distancing). Even more, several countries imposed long-lasting curfews, one of the longest (174 days) in Slovenia, limiting or eliminating time for outdoor activities. Although the restrictions help to limit the spread of the virus, they also impose a direct and indirect burden on public health.

Globally, a multicentric 22-country study revealed that the COVID-19 home confinement has had a medium to large (23–43%) negative effect on all levels of PA (vigorous, moderate, walking and overall) and a large increase in daily sitting time by more than 28% in adults (1). However, effects of COVID-19 lockdown on children were even greater than on adolescents and adults or seniors (2) where authors reported a 42% decreased in PA, a 36% increase in sitting time and a 62% increase in screen time for children.

PA (in combination with sedentary behavior) is associated with numerous health benefits for children and adolescents, including cardiometabolic health, motor skill development, bone density, and emotional regulation/psychological health (3, 4). However, even prior to the COVID-19 pandemic, <10% of school aged (5–17 years) children achieved recommended amounts of physical PA (5). Since the onset of the COVID-19 pandemic only 4.8% of Canadian children were meeting PA guidelines (6). Furthermore, there are limited data investigating changes in children's PA levels during COVID-19 pandemic compared to before pandemic. Among children PA levels decreased, and screen time increased by 52 and 145% in Spain (7), by 32 and 54% in Germany (8) by 64 and 175% in Italian children (9), respectively.

During 2020 an estimated 1.5 billion children (age 5–12 years old) and adolescents (age 13–17 years old) transitioned to remote schooling following school closures or partially closures (10). During the 2nd wave of the COVID-19 pandemic, Slovenian primary schools were closed from October 19th 2020, until January 18th 2021 when they were partially opened for 6–9 year olds and from February 15th 2021 for all children. In total, Slovenian children did not attend school for 89 calendar days or 60 working days. In parallel, all curricular and extracurricular sports programs were completely closed for the same period, while most of sport programs were closed for the entire 2020/21 school year.

Despite the benefits of PA on child health and wellbeing, new data suggest that during COVID-19 pandemic, access to health-promoting PA has been largely disrupted, whereas use of screen-based media for education and recreation has increased (11–14) when compared to studies from before the COVID-19 pandemic (15, 16). Important here is to know that short-term changes in PA and sedentary behavior in reaction to COVID-19 may become permanently entrenched, leading to increased risk of obesity, diabetes, and cardiovascular disease in children (11). During the COVID-19 restrictions, USA children spent 90 min in school-related sitting with over 8 h of leisure time sitting, where greater decrease in PA and increase in sitting time was reported in adolescents when compared to younger children (11). Additionally, school physical education (PE) represents the largest youth PA intervention worldwide. And school closures due to COVID-19 created a new set of obstacles as PE shifted from playgrounds and gyms to virtual learning platforms (17). Despite the considerable amount of research, especially on general effects on children's PA, physical inactivity and sedentary behavior associated with screen time, the little is known about the implementation of PE (on-line)clasess in the period of COVID-19 restrictions. Compering PE implementation time with a comprehensive analysis of school-based and extracurricular physical/sport activity in children before and during the COVID-19 restriction is still a missing gap in the field of study.

Increased physical inactivity level is not only regularly associated with increased risk of severe health outcomes in adults, but also with adiposity, lipid profile, insulin and glucose levels, blood pressure and other cardiovascular risks in children (18). Furthermore, it is also associated with serious COVID-19 outcomes in adults (19). Therefore, it would be of great interest to limit the level of physical inactivity already in children, especially if it occurs regularly in secular trends and during COVID-19 pandemics.

The aim of the study is to evaluate the changes in curricular physical education (PE) and leisure PA during COVID-19 restrictions compared to period before. Specifically, we focused on the implementation of PE in schools, extracurricular sports activities organized by schools, organized activities in sports clubs, and non-organized PA with friends, family or individually.

## Materials and Methods

### Data Collection From the Questionnaire Survey

An on-line survey was created about PA, quality of life, and eating habits of primary school students. The survey was intended for parents of second to fifth grade students aged 6 to 12 years (mean = 8.81, SD = 1.46) in Slovenia. The time frame of the survey refers to the period before (BEFORE) the outbreak of the epidemic (March 12, 2020) and the imposed measures to contain the COVID-19 epidemic as well as to the period when the transition to remote schooling was ordered at the state level (DURING). The survey was conducted in the period from 23 December 2020 to 15 January 2021, i.e., for a total duration of 24 days. An invitation to participate in the survey with a detailed explanation for school principals and parents, was sent to all primary school in Slovenia by the Director-General of the Directorate for Preschool and Primary Education of the Ministry of Education, Science and Sport. The research group also sent the invitation directly to primary schools. Parents as the target group were invited through a circular letter from the of Slovenian primary school principals, while it was also the task of classroom teachers to invite parents to participate.

During the period up to 15 January 2021, 5,282 clicks were received in response to the invitation and 4,737 (90%) parents and guardians chose to complete the survey, representing a high response rate. Three thousand nine hundred and thirty-six (74%) questionnaires completed for PA domain, were accepted for further analysis.

Participation in the online survey was voluntary and respondents could opt out at any time before the survey was completed. By clicking on survey, parents and guardians agreed to participate and additionally received additional instructions on how to complete the survey.

### Survey Questionnaires

The questionnaire entitled “CHILDREN AND MEASURES DURING THE COVID-19 EPIDEMIC” comprises of 58 variables and 29 questions divided in four sets, i.e.,:

- The socio-demographic section to collect basic demographic data of children, related to the current status of the child and the family;
- The standardized International Physical Activity Questionnaire—Short form (IPAQ-SF) (20). For the needs of the survey, the IPAQ-SF questionnaire was translated by a certified translator and slightly amended for the needs of the survey to collect parents' assessments of the child's PA. Specifically, we added questions to collect data on the child's inclusion and implementation of physical education (PE); extracurricular, school- and sport club-organized sport activities; and non-organized PA (with friends, family or individually). All questions were referred to BEFORE and DURING lockdown, meaning that every question required two answers. Responses related to the duration of PA were defined in terms of categorical ordinal variables as labeled in row/column labels in **Tables 2**–**10**. Such formulation of answers facilitated parents' responses regarding the duration of PA, as parents are not with their children all day and therefore it is difficult to estimate the duration in minutes. Therefore, only frequencies were reported and analyzed for each variable.
- Standardized KINDL–R (Questionnaire for Measuring Health-Related Quality of Life in Children and Adolescents), (21). The parents' version of the questionnaire was selected to meet the needs of the project (1.2.2 Parents' versions KINDL). This standardized questionnaire was also translated from English into Slovenian by a certified translator. Back translation was also done to ensure the validity of the translation. Data on these are not included in this report.
- Questionnaire on the child's eating habits, with questions on frequency of consumption of unhealthy and healthy food and beverages as well as eating habits and regular meals, again for the period before and during remote schooling. Data on these are not included in this report.

### Data Privacy

Parents and guardians who responded to the survey, were informed in the introductory section that all data would be processed and managed in compliance with the provisions of the applicable legislation on the protection of personal data and the General Data Protection Regulation (GDPR). Respondents' answers were anonymous and confidential and are kept for research purposes in the archives of the survey providers, the Faculty of Education of the University of Maribor and the Science and Research Centre Koper. The survey questionnaire does not contain any personal data (name, date of birth or other contact information) that would allow the respondents identification. It also does not contain any ethically or morally disputable questions.

### Statistical Analysis

The data from the questionnaire were processed with SPSS, version 21. Descriptive statistics were calculated (mean, standard deviation, frequency). The χ^2^ test was carried out to analyse differences in all PA variables BEFORE and DURING lockdown. The level of significance was set at *p* < 0.05.

## Results

### Sample Description

The questionnaire on PA was completed in full by parents and guardians of 3,936 children; of these 2,070 (52.6%) were boys and 1,866 (47.4%) were girls. The age range of children was from 6 to 12 years (mean = 8.81 years, SD = 1.46). Respondents came from all 12 Slovenian regions. The detailed data are presented in Table 1.

**Table 1:** Demographic characteristics of the participants.

**Variable**	**Demographic characteristics**	**Frequency**	**%**
Gender	Boys	2,070	52.6
	Girls	1,866	47.4
Age (years)	6	79	2.0
	7	781	19.8
	8	939	23.9
	9	829	21.1
	10	870	22.1
	11	191	4.9
	12	247	6.3
Number of household members	2	111	2.8
	3	668	17.0
	4	2,108	53.6
	5	753	19.1
	6	219	5.6
	>6	77	2.0
Region	1. Upper Carniola region	259	6.6
	2. Gorizia region	276	7.0
	3. Southeast Slovenia region	354	9.0
	4. Carinthia region	146	3.5
	5. Coastal-Karst region	210	5.3
	6. Central Slovenia region	895	22.7
	7. Drava region	489	12.4
	8. Mura region	343	8.7
	9. Lower Sava region	153	3.9
	10. Littoral-Inner Carniola region	216	5.5
	11. Savinja region	433	11.0
	12. Central Sava region	162	4.1

### Physical Activity

In Slovenia, PE takes place three times a week in primary school, for the age group of children in the selected sample. The duration of a single PE session is 45 min and one of its objectives is to encourage children to engage/to achieve moderate to vigorous physical activity (MVPA). DURING lockdown, PE was implemented in a variety of ways. Teachers provided online instructions to students via text or videos, while PE sessions were also delivered live, via video call. The results in Table 2 show that 38.6% of PE session lasted < 15 min, 39.1% lasted from 16 to 30 min, 18% from 31 to 44 min, and only 4.3% of students exercised ≥45 min, which is also the learning objective. This means that overall, of 95.7% of the students had a substantially shorter duration of PE sessions DURING lockdown than in the period BEFORE lockdown.

**Table 2:** Duration of an individual session of PE during lockdown.

**Duration of exercise session**	**Frequency**	**%**
<15 min	1,520	38.6
16–30 min	1,538	39.1
31–44 min	708	18.0
≥45 min	170	4.3
Total	3,936	100

Table 3 shows a decline in participation in school extracurricular sports activities BEFORE and DURING lockdown (χ^2^ = 383.2; *p* < 0.001) after grouping DURING data in two categories: never and ≥1 session (grouping categories: 1 session, 2 sessions, 3 sessions, >3 sessions). In particular, 72.2% of children participated in at least one school extracurricular sports activity BEFORE, while DURING 83.5% of them did not. BEFORE, 21.8% of children participated in one session per week and DURING lockdown this proportion decreased to 7.8%. BEFORE lockdown, 26% of children participated two sessions, while DURING lockdown only 4.1% had two sessions per week. BEFORE lockdown, 14.6% of children participated in three sessions a week and DURING lockdown only 2.9% continued this practice. 9.8% of children had more than three weekly sessions BEFORE lockdown and DURING lockdown only 1.7% remained included/persisted.

**Table 3:** Contingency table for comparison of frequency (category percentages for during; category percentages for before) of participation in school extracurricular sports activities before and during lockdown.

		**DURING**						
		**Never**	**1 session**	**2 sessions**	**3 sessions**	**>3 sessions**	**Total ≥1 sessions**	**Total**
BEFORE	Never	1,078 (32.8; 98.6)	4 (1.3; 0.4)	8 (5.0; 0.7)	3 (2.6; 0.3)	0 (0; 0)	15 (2.3; 1.4)	1,093 (27.8; 100)
	1 session	721 (21.9; 83.8)	115 (37.3; 13.4)	12 (7.5; 1.4)	6 (5.2; 0.7)	6 (9.2; 0.7)	139 (21.4; 16.2)	860 (21.8; 100)
	2 sessions	846 (25.7; 82.5)	85 (27.6; 8.3)	75 (46.6; 7.3)	12 (10.3; 1.2)	7 (10.8; 0.7)	179 (27.5; 17.5)	1,025 (26.0; 100)
	3 sessions	401 (12.2; 70.0)	63 (20.5; 11.0)	40 (24.8; 7.0)	59 (50.9; 10.3)	10 (15.4; 1.7)	172 (26.5; 30.0)	573 (14.6; 100)
	>3 sessions	240 (7.3; 62.3)	41 (13.3; 10.6)	26 (16.1; 6.8)	36 (31.0; 9.4)	42 (64.6; 10.9)	145 (22.3; 37.7)	385 (9.8; 100)
	Total	3,286 (100; 83.5)	308 (100; 7.8)	161 (100; 4.1)	116 (100; 2.9)	65 (100; 1.7)	650 (100; 16.5)	3,936 (100; 100)

*χ^2^ = 383.2; p < 0.001*.

Table 4 shows the differences in duration of an individual/single session in extracurricular sport activities between BEFORE and DURING lockdown (χ^2^ = 267.0; *p* < 0.001). The duration of individual sessions mainly decreased. BEFORE lockdown, 68% of children engaged in sessions of duration ≤45 min and DURING lockdown this proportion increased to 79.0%. BEFORE, 19.7% of children participated in sessions 46–60 min long and DURING lockdown this percentage fell to 14.7%. Sessions longer than 60 min BEFORE lockdown were reported by 12.3% of the respondents, while for the period DURING lockdown this percentage fell to only 6.2%.

**Table 4:** Contingency table for comparison of frequency (category percentages for during; category percentages for before) of duration of an individual session of extracurricular sport activities before and during lockdown.

		**DURING**			
		**≤45 min**	**46–60 min**	**>60 min**	**Total >0 min**
BEFORE	≤ 45 min	445 (79.7; 92.7)	24 (23.1; 5.0)	11 (25.0; 2.3)	480 (68.0; 100)
	46–60 min	74 (13.3; 53.2)	61 (58.7; 43.9)	4 (9.1; 2.9)	139 (19.7; 100)
	>60 min	39 (7.0; 44.8)	19 (18.3; 21.8)	29 (65.9; 33.3)	87 (12.3; 100)
	Total	558 (100; 79.0)	104 (100; 14.7)	44 (100; 6.2)	706 (100; 100)

*χ^2^ = 267.0; p < 0.001*.

Table 5 shows a major decline in the participation in MVPA in sports clubs BEFORE and DURING lockdown (χ^2^ = 302.8; *p* < 0.001) after grouping DURING data in two categories: never and ≥1 session (grouping categories: 1 session, 2 sessions, 3 sessions, >3 sessions). We found that BEFORE lockdown, 69.7% of children participated in at least one session weekly. In the period DURING, this changed substantially, as 88.1% did not engage in such activities at all. BEFORE, 15.7% of children had one session and in the period DURING this percentage fell to 3.9%. BEFORE lockdown, 28.4% of children participated in two sessions, while DURING lockdown only 5% had two sessions a week. In the period BEFORE, 14.6% of children participated in three sessions a week and in the period DURING, this percentage stood at 1.8%. BEFORE lockdown, 9.8% of children participated in more than three sessions, while DURING lockdown only 1.3% had three sessions a week.

**Table 5:** Contingency table for comparison of frequency (category percentages for during; category percentages for before) of participation in sports clubs before and during lockdown.

		**DURING**						
		**Never**	**1 session**	**2 sessions**	**3 sessions**	**>3 sessions**	**Total ≥1 sessions**	**Total**
BEFORE	Never	1,183 (34.1; 99.3)	5 (3.3; 0.4)	0 (0; 0)	1 (1.4; 0.1)	2 (4.0; 0.2)	8 (1.7; 0.7)	1,191 (30.3; 100)
	1 session	558 (16.1; 90.6)	50 (37.3; 13.4)	5 (7.5; 1.4)	2 (5.2; 0.7)	1 (9.2; 0.7)	58 (12.3; 9.4)	616 (15.7; 100)
	2 sessions	948 (27.4; 84.8)	49 (32.0; 4.4)	109 (55.9; 9.7)	8 (11.1; 0.7)	4 (8.0; 0.4)	170 (36.2; 15.2)	1,118 (28.4; 100)
	3 sessions	509 (12.2; 70.0)	30 (20.5; 11.0)	49 (24.8; 7.0)	35 (50.9; 10.3)	8 (15.4; 1.7)	122 (19.3; 26.0)	631 (14.6; 100)
	>3 sessions	268 (7.7; 70.5)	19 (12.4; 5.0)	32 (16.4; 8.4)	26 (36.1; 6.8)	35 (70.0; 9.2)	112 (23.8; 29.5)	380 (9.8; 100)
	Total	3,466 (100; 88.1)	153 (100; 3.9)	195 (100; 5.0)	72 (100; 1.8)	50 (100; 1.3)	650 (100; 12.0)	3,936 (100; 100)

*χ^2^ = 302.8; p < 0.001*.

The results in Table 6 show the difference in duration of an individual/single session in sports clubs BEFORE and DURING lockdown (χ^2^ = 274.0; *p* < 0.001; due to the low values of individual frequencies, the χ^2^ Likelihood Ratio has been used in some cases). BEFORE lockdown, 3.4% of children engaged in sessions of duration ≤30 min and DURING lockdown this proportion increased to 14.5%. BEFORE 8.5% of children participated in sessions lasting 31–45 min and DURING lockdown this percentage stood at 20.3% of children. Sessions lasting 40–60 min BEFORE lockdown were reported by 36.2% of the respondents, while for the period DURING lockdown this percentage stood at 39%. Sessions lasting longer than 60 min BEFORE lockdown were reported by 51.8% of respondents, while this percentage for the period DURING lockdown was only 26.2%. To summarize, the longer the duration of the session, the smaller the proportion of the children DURING compared to BEFORE.

**Table 6:** Contingency table for comparison of frequency (category percentages for during; category percentages for before) of duration of an individual session in sports clubs before and during lockdown.

		**DURING**				
		**≤30 min**	**31–45 min**	**46–60 min**	**>60 min**	**Total**
BEFORE	≤ 30 min	15 (22.1; 93.8)	1 (1.1; 6.3)	0 (0)	0 (0)	16 (3.4; 100)
	31–45 min	9 (13.2; 22.5)	29 (30.5; 72.5)	2 (1.1; 5.0)	0 (0)	40 (8.5; 100)
	46–60 min	18 (26.5; 10.6)	30 (31.6; 17.6)	117 (63.9; 68.8)	5 (4.1; 2.9)	170 (36.2; 100)
	>60 min	26 (38.2; 10.7)	35 (36.8; 14.4)	64 (35.0; 26.3)	118 (95.9; 48.6)	243 (51.8; 100)
	Total	68 (100; 14.5)	95 (100; 20.3)	183 (100; 39.0)	123 (100; 26.2)	469 (100; 100)

*χ^2^ (Likelihood Ratio) = 274.0; p < 0.001*.

Table 7 shows differences in the participation in non-organized MVPA between BEFORE and DURING lockdown (χ^2^ = 967.0; *p* < 0.001) after grouping DURING data in two categories: never and ≥ 1 session (grouping categories: 1 session, 2 sessions, 3 sessions, >3 sessions). BEFORE lockdown, 8.2% of children did not participate in such sessions, while in the period DURING lockdown this proportion increased to 24.9%. BEFORE, 18.6% of children participated in one session per week and DURING lockdown this proportion increased to 20.2%. BEFORE lockdown, 23.6% of children participated in two sessions, while DURING lockdown this proportion decreased to 17.8%. In the period BEFORE, 17.4% of children participated in three sessions per week and in the period DURING, this percentage stood at 12.1%. BEFORE lockdown, 32.3% of children participated in more than three sessions and DURING lockdown this proportion fell to 24.8%. The results show that the proportion of active children in non-organized MVPA BEFORE and DURING slightly decreased, however significantly less than in organized sports activities.

**Table 7:** Contingency table for comparison of frequency (category percentages for during; category percentages for before) of participation in non-organized moderate-to-vigorous physical activity before and during lockdown.

		**DURING**						
		**Never**	**1 session**	**2 sessions**	**3 sessions**	**>3 sessions**	**Total ≥1 sessions**	**Total**
BEFORE	Never	296 (30.1; 91.9)	13 (1.6; 4.0)	8 (1.1; 2.5)	1 (0.2; 0.3)	4 (0.4; 1.2)	26 (0.9; 8.1)	322 (8.2; 100)
	1 session	212 (21.6; 29.0)	316 (39.6; 43.2)	107 (15.3; 14.6)	64 (13.4; 8.8)	32 (3.3; 4.4)	519 (17.6; 71.0)	731 (18.6; 100)
	2 sessions	236 (24.0; 25.4)	198 (24.8; 21.3)	276 (39.4; 29.7)	88 (18.4; 9.5)	131 (13.4; 14.1)	693 (23.5; 74.6)	929 (23.6; 100)
	3 sessions	123 (12.5; 18.0)	136 (17.1; 19.9)	117 (16.7; 17.1)	219 (45.8; 32.0)	89 (9.1; 13.0)	561 (19.0; 82.0)	684 (17.4; 100)
	>3 sessions	115 (11.7; 9.1)	134 (16.8; 10.6)	193 (27.5; 15.2)	106 (22.2; 8.3)	722 (73.8; 56.9)	1,155 (39.1; 90.9)	1,270 (32.3; 100)
	Total	982 (100; 24.9)	797 (100; 20.2)	701 (100; 17.8)	478 (100; 12.1)	978 (100; 24.8)	2,954 (100; 74.9)	3,936 (100; 100)

*χ^2^ = 967.0; p < 0.001*.

Table 8 shows difference in the duration of an individual session in non-organized MVPA between BEFORE and DURING lockdown (χ^2^ = 2343.6; *p* < 0.001). In general, the duration of individual sessions slightly decreased. BEFORE lockdown, 10.8% of children engaged in sessions of duration ≤ 30 min and DURING lockdown this proportion increased to 21.2%. Sessions in duration of 31–45 min BEFORE lockdown were reported by 25.1% of the respondents, while this percentage for the period DURING lockdown stood at 24.7%. BEFORE, 32.7% of children participated in sessions lasting 46–60 min and DURING lockdown this percentage stood at 27.9%. Sessions lasting longer than 60 min BEFORE lockdown were reported by 31.4% of the respondents, while this percentage for the period DURING lockdown was at 26.2%.

**Table 8:** Contingency table for comparison of frequency (category percentages for during; category percentages for before) of duration of an individual session of non-organized moderate-to-vigorous physical activity before and during lockdown.

		**DURING**				
		**≤30 min**	**31–45 min**	**46–60 min**	**>60 min**	**Total**
BEFORE	≤ 30 min	249 (38.5; 75.5)	48 (6.4; 14.5)	24 (2.8; 7.3)	9 (1.1; 2.7)	330 (10.8; 100)
	31–45 min	174 (26.9; 22.7)	449 (59.7; 58.7)	108 (12.7; 14.1	34 (4.3; 4.4)	765 (25.1; 100)
	46–60 min	136 (21.0; 13.7)	157 (20.9; 15.8)	574 (67.5; 57.6	129 (16.2; 13.0)	996 (32.7; 100)
	>60 min	88 (13.6; 9.2)	98 (13.0; 10.3)	145 (17.0; 15.2)	625 (78.4; 65.4)	956 (31.4; 100)
	Total	647 (100; 21.2)	752 (100; 24.7)	851 (100; 27.9)	797 (100; 26.2)	3,047 (100; 100)

*χ^2^ = 2,343.6; p < 0.001*.

Table 9 shows differences in the participation in non-organized light physical activity (LPA) between BEFORE and DURING lockdown (χ^2^ = 689.7; *p* < 0.001) after grouping DURING data in two categories: never and ≥1 session (grouping categories: 1 session, 2 sessions, 3 sessions, >3 sessions). We found that BEFORE lockdown, 95.2% of children participated in at least one session a week. DURING lockdown, the percentage of such children stood at 87%. BEFORE, 16.7% of children participated in one session a week and in the period DURING, this percentage stood at 16.1%. BEFORE lockdown, 24.5% of children participated in two sessions and DURING lockdown only 19.1% had two sessions a week. BEFORE, 17.7% of children had three sessions a week and in the period DURING this percentage stood at 16.4%. BEFORE lockdown, 36.3% of children participated in more than three sessions, while DURING lockdown this percentage stood at 35.4%.

**Table 9:** Contingency table for comparison of frequency (category percentages for during; category percentages for before) of participation in non-organized light physical activity before and during lockdown.

		**DURING**						
		**Never**	**1 session**	**2 sessions**	**3 sessions**	**>3 sessions**	**Total ≥1 sessions**	**Total**
BEFORE	Never	137 (26.9; 73.3)	17 (2.7; 9.1)	14 (1.9; 7.5)	9 (1.4; 4.8)	10 (0.7; 5.3)	50 (1.5; 26.7)	187 (4.8; 100)
	1 session	97 (19.0; 14.8)	257 (40.5; 39.2)	110 (14.6; 16.8)	94 (14.6; 14.3)	98 (7.0; 14.9)	559 (16.3; 85.2)	656 (16.7; 100)
	2 sessions	137 (26.9; 14.2)	150 (23.7; 15.5)	371 (49.3; 38.4)	116 (18.0; 12.0)	192 (13.8; 19.9)	829 (24.2; 85.8)	966 (24.5; 100)
	3 sessions	63 (12.4; 9.0)	101 (15.9; 14.5)	110 (14.6; 15.8)	289 (44.8; 41.4)	135 (9.7; 19.3)	635 (18.5; 91.0)	698 (17.7; 100)
	>3 sessions	76 (14.9; 5.3)	109 (17.2; 7.6)	147 (19.5; 10.3)	137 (21.2; 9.6)	960 (68.8; 67.2)	1,353 (39.5; 94.7)	1,429 (36.3; 100)
	Total	510 (100; 13.0)	634 (100; 16.1)	752 (100; 19.1)	645 (100; 16.4)	1,395 (100; 35.4)	3,426 (100; 100)	3,936 (100; 100)

*χ^2^ = 689.7; p < 0.001*.

The results in Table 10 show difference in the duration of non-organized LPA between BEFORE and DURING lockdown (χ^2^ = 3145.9; *p* < 0.001). BEFORE lockdown, 12.3% of children engaged in sessions of duration ≤ 30 min and DURING lockdown this proportion increased to 17.9%. BEFORE, 26.2% of children participated in sessions lasting 31–45 min and DURING lockdown this percentage stood at 24.7%. Sessions lasting 40–60 min BEFORE lockdown were reported by 31.2% of respondents, while this percentage for the period DURING lockdown was at 27.9 %. Sessions lasting longer than 60 min BEFORE lockdown were reported by 30.4% of the respondents, while this percentage for the period DURING lockdown stood at 29.5%.

**Table 10:** Contingency table for comparison of frequency (category percentages for during; category percentages for before) of duration of an individual session of non-organized light physical activity before and during lockdown.

		**DURING**				
		**≤30 min**	**31-45 min**	**46-60 min**	**>60 min**	**Total**
BEFORE	≤ 30 min	304 (50.2; 73.3)	56 (6.7; 13.5)	38 (4.0; 9.2)	17 (1.7; 4.1)	415 (12.3; 100)
	31–45 min	129 (21.3; 14.6)	553 (66.4; 62.2)	136 (14.4; 15.4)	66 (6.6; 7.5)	884 (26.2; 100)
	46–60 min	105 (17.4; 10.0)	134 (16.1; 12.7)	653 (69.2; 62.0)	161 (16.2; 15.3)	1,053 (31.2; 100)
	>60 min	67 (11.1; 6.5)	90 (10.8; 8.8)	116 (12.3; 11.3)	752 (75.5; 73.4)	1,025 (30.4; 100)
	Total	605 (100; 17.9)	833 (100; 24.7)	943 (100; 27.9)	996 (100; 29.5)	3,377 (100; 100)

*χ^2^ = 3,145.9; p < 0.001*.

## Discussion

The purpose of this study was to examine how the restrictions, which were imposed due to COVID-19 pandemic, have been reflected in the implementation of the different forms of PA of children aged 6–12 years. We found that DURING lockdown, PE was implemented by teachers providing students with online instructions, in the form of text or videos, while PE sessions were also held live, via video call. The fact is that shift to remote schooling was a major challenge for teachers. At the same time, it was also unclear which strategies were the most effective for the remote implementation of PE. The results of our study show that a total of 95.7% of students had a substantially shorter implementation of PE sessions DURING lockdown than in the period BEFORE lockdown. Worryingly, 38.6% of students engaged in PE sessions of <15 min and as many as 77.7 % of children performed PE sessions of lasting ≤30 min DURING lockdown. This was much less than required by school curriculum (45 min).

A significant decline was also noted in children's participation in school extracurricular sports activities. In the period BEFORE lockdown, 72.2% of children participated in at least one school extracurricular sports activity a week. In the period DURING lockdown, 83.5% of children did not participate in any such activity. There was a decrease in the weekly number of sessions in school extracurricular sports activities as well as a decline in the duration of individual sessions. A decrease of children' participation in sports sessions held in sports clubs was noted. BEFORE lockdown, 69.7% of children attended at least one session weekly. In the period DURING, as many as 88.1% of children did not participate in such activities at all. The weekly number of these sessions per child and the duration of a single session also decreased. The situation is similar, though slightly better, in non-organized MVPA. We found that BEFORE lockdown, 8.2% of children did not engage in this type of activity, while this percentage DURING lockdown stood at 24.9%. The duration of individual sessions in non-organized MVPA also declined slightly in the period DURING compared to the period BEFORE lockdown. The results show that the proportion/percentage of active children in non-organized MVPA BEFORE slightly decreased, however much less than in organized sports activities. The same also applies to the duration of individual/single sessions. We found that BEFORE lockdown, 95.2% of children participated in at least one session of non-organized LPA a week. DURING lockdown, the percentage of such children was 87%. The duration of individual sessions was slightly longer BEFORE compared to the DURING lockdown period. Again, it was found that there were no major differences between the period BEFORE and DURING lockdown.

To date, no research has been observed which address the frequency and duration of PE in schools, extracurricular sports activities organized by schools, organized activities in sports clubs, and non-organized PA for the COVID-19 lockdown period. However, the results of our study are consistent with other individual studies that found that COVID-19 lockdown had a major negative effect on all PA intensity levels, while also increasing daily sitting time (1, 22). Genin et al. (2) found that the effects of COVID-19 lockdown on children were even greater than those on adolescents and adults. In Canada, an international survey (6) showed that during lockdown a very small proportion of children met PA recommendations. At the same time, the decrease in PA was paralleled to increased sitting and screen time (9). Ding et al. (23) report on the COVID-19 lockdown which led to a substantial decline of PA levels in 11 countries across the globe. The authors thus recommend that countries implement PA promotion interventions. Another study found a substantial decrease in PA levels during leisure time, PE, and recess of Czech children during the COVID-19 lockdown (24). During the COVID-19 lockdown, older students in Norway recorded higher PA levels compared to younger students (25). The authors raise the questions of whether older students were given too much responsibility for their own PA during this period and whether teachers should offer students more remote workout sessions using digital technologies.

An increase in physical inactivity, or a decrease in PA, during the COVID-19 epidemic was also reported by Bu et al. (26). The authors believe that given the well-established relationship between PA and health, an increase in physical inactivity will have both immediate and long-term implications for people's physical and mental health and general wellbeing. In this respect, the authors state that more efforts are needed to promote PA during the pandemic and beyond. In their systematic review, Stockwell et al. (27) noted that a majority of studies report a decrease in PA and an increases in sedentary behaviors during lockdown across several populations, including children. The authors believe that public health strategies should include the development and implementation of interventions that promote safe PA and reduce sedentary behavior if further lockdowns occur.

Future intervention measures to contain the COVID-19 epidemic should take in to account the findings that lockdowns have negative effects, by drastically limiting the participation in non-organized and organized PA and access to numerous types of activities (e.g., closed gyms, no group meetings, social distancing). In addition to all the known negative consequences of low PA, recent studies also show that physical inactivity in adults is associated with a higher risk of severe COVID-19 outcomes (19). As great effort has been made in the last 20 years to reduce obesity and increase the fitness level of children and adolescents, a drastic decline in all fitness dimensions (SLOfit test battery) was observed after COVID-19 restrictions (28). The decline was so severe, that the average Slovenian child's overall locomotor efficiency decrease by more than 13% after only 2-months COVID-19 restrictions. Therefore, our findings allow us to suggest that incentives for regular PA and reduction of physical inactivity (sedentarism) during COVID-19 restricted period should be classified as one of the most effective strategies for short- and long-term prevention of public health.

These findings are a good starting point for shaping an effective strategy to promote a health-enhancing children's PA in the event of repeated pandemic restrictions, or otherwise for the implementation of organized forms of in-person sports activities. The strategy should predominantly/primarily focus on motivating children to participate in organized and non-organized regular PA for an average of at least of 60 min per day and limiting sedentary time (especially screen time), in line with the WHO guidelines from 2020. Motivation is even more important, as teachers in remote education have no face-to-face contact with students, limited possibility for interaction, difficulty in corrective work of movement techniques. It is particularly important to motivate groups of children who are otherwise already not physically active enough. In the light of the obtained results, it can be recommended that, much more emphasis should be placed on organized sport activities in schools and sports clubs in the event of another lockdown. Practical experience has shown that sport activities can be carried out successfully implemented remotely if is not possible to carried them face-to-face. All in all, organized sport activities could also be implemented face-to-face, considering professional recommendations (29). In any event, it is necessary to take a different approach to the implementation of organized sport activities than during the second wave of the epidemic which lasted from autumn 2020 to spring 2021. The strategy should primarily focus on motivating young people and on providing organized activities that are professionally run, sufficiently frequent, and appropriate in terms of time. The smallest decline was observed in non-organized PA, which, in the case of children, usually takes place within the family. However, here too, the strategy should be geared toward promoting PA, especially in nature, to replace screen time and especially recreational screen time (30).

## Strengths and Limitations

The most important strength of this study is the large sample of respondents, covering parents and guardians of children from all Slovenian regions. This allows us to generalize the obtained results to the entire population of Slovenian children. The limitations of the study mainly refer to the fact that the questionnaire was answered only by parents and guardians, although this was the only way, as they are not with children all day, so it is difficult for them to exactly define the duration and the intensity of their child's PA.

## Data Availability Statement

The original contributions presented in the study are included in the article/supplementary material, further inquiries can be directed to the corresponding author/s.

## Ethics Statement

The studies involving human participants were reviewed and approved by Ethical Committee of the Science and Research Centre Koper, Slovenia (No. 0624-13/21). The patients/participants provided their written informed consent to participate in this study.

## Author Contributions

JP, ČM, SP, RP, and BŠ contributed to the study design and execution, writing the manuscript, and provided critical feedback. ČM and SP carried out all primary data collection. All authors reviewed the results and approved the final version of the manuscript.

## Funding

This study was conducted along with the research program Kinesiology for Quality of Life and its upgrade by research topic Empirical Research and Modeling of Human Behavior During Social Distancing Measures, both financed by the Slovenian Research Agency. The study was coordinated by the researchers of the Institute for Kinesiology Research at Science and Research Centre of Koper and Faulty of Education at University of Maribor.

## Conflict of Interest

The authors declare that the research was conducted in the absence of any commercial or financial relationships that could be construed as a potential conflict of interest.

## Publisher's Note

All claims expressed in this article are solely those of the authors and do not necessarily represent those of their affiliated organizations, or those of the publisher, the editors and the reviewers. Any product that may be evaluated in this article, or claim that may be made by its manufacturer, is not guaranteed or endorsed by the publisher.
